# A Young Iranian Woman with Pure Primary Ovarian Neuroendocrine Tumor: A Case Report

**DOI:** 10.30699/IJP.2022.542788.2766

**Published:** 2021-08-14

**Authors:** Fatemeh Samiee-Rad, Mahdi Ghaebi, Arezoo Bajelan

**Affiliations:** Clinical Research Development Unit, Kosar Hospital, Qazvin University of Medical Sciences, Qazvin, Iran

**Keywords:** Neuroendocrine tumor, Neuroendocrine neoplasm, Ovary, Primary, Pure

## Abstract

Pure ovarian neuroendocrine tumors are very rare. Herein, we present a young Iranian woman with a pure primary ovarian neuroendocrine tumor.

A 26-year-old female presented with chronic abdominal pain and progressive constipation and was referred to the emergency room. Imaging findings confirmed a mass in the right adnexa. Following the resectional surgery of the ovarian mass, histopathological and immunohistochemistry results disclosed a mixed type of primary ovarian neuroendocrine tumor. The patient did not experience tumor recurrence afterward.

Due to the rarity and low prevalence of primary pure ovarian neuroendocrine tumors, the histopathologic diagnosis should be confirmed by an immunohistochemistry study.

## Introduction

Carcinoid tumors (CT), recently known as neuroendocrine tumors (NETs), derive from neuroendocrine cells show capability for secreting a variety of neuropeptides, causing clinical symptoms. NETs usually originate from the gastrointestinal or respiratory tract and rarely from other organs, including ovaries, and generally account for less than 0.5% of all malignancies (1). Neuroendocrine markers are the key features to define the origin of NETs at any anatomic site (2). 

Ovarian NETs are rare, accounting for less than 5% of all NETs and 0.1% of all ovarian tumors (3). The prevalence of genitourinary NETs has been generally estimated as about 7% of all NETs in the country of study (1). Almost all ovarian NETs are classified as germ cell tumors, often combined with other teratomatous components, and only less than 15% of them are found in pure form (3). They can be primary or metastatic, mostly from the GI tract, and histomorphological are divided into insular, trabecular, stromal, mucinous and mixed carcinoids subtypes. Ovarian mixed NETs are a combination of the mentioned subtypes with each other or with other ovarian tumors, such as epithelial neoplasms or sex cord stromal tumors (4). NETs are classified as neuroendocrine tumors (NETs) G1–3, neuroendocrine carcinoma (NEC), small-cell type (SCNEC) and large-cell type (LCNEC), based on mitotic activity and Ki67 index (5). 

Ovarian NETs most commonly appear as a unilateral adnexal mass in patients around 50 to 70 years of age and represent a carcinoid syndrome episode (6). Carcinoid syndrome is a paraneoplastic syndrome associated with less than 30% of NETs, especially larger well differentiated tumors, and is caused by the systemic release of different hormonal factors (7). Constipation is an uncommon symptom of carcinoid syndrome caused by the production of YY peptide, a gastrointestinal hormone that decreases gut motility (8). 

Radiologically, ovarian NETs commonly appear as solid cystic masses (3, 9) and can arise from different teratomatous components, requiring intent IHC staining (10). Chromogranin A (CgA) and synaptophysin are the most specific markers for ovarian NETs (10). PAX8 is another important IHC marker with high sensitivity and specificity for detecting the Mullerian origin of CTs (11). 

We report a rare primary pure ovarian NET in a 26-year-old Iranian woman suffering from chronic abdominal pain and chronic progressive constipation as a scarce symptom of carcinoid syndrome. She was admitted to the emergency room with acute abdominal pain due to the torsion of the ovary, despite the small size of the tumor. Before enrolling the patient in this study, participant consent was obtained, and all applicable international, national and/or institutional guidelines were followed.

## Case Presentation

The patient was a 26-year-old woman, with a history of two pregnancies with the outcome of two live children, without any family history or personal risk factors for malignancy. She suffered from chronic abdominal pain with chronic progressive constipation for six months and was admitted to the emergency of Kosar academic hospital (Qazvin, Iran) in April 2018, with a chief complaint of lately intensified abdominal pain in the right lower quadrant region, accompanied by nausea and anorexia. A tender pelvic mass in the right adnexa was palpated. Nevertheless, appendicitis was ruled out by specific physical examinations.

Sonographic studies showed a right adnexal mass, measured 64 × 54 ×54 millimeters. In computerized tomography (CT) scan, a well-defined solid cystic mass was seen in the right adnexa, M: 63 ×53 ×53 mm, with echogenic foci inside the solid part and no invasion of the surrounding tissue were detected. Subserosal leiomyoma was considered as the most likely possibility in differential diagnosis. Laboratory studies showed exclusively mild leukocytosis, while other laboratory studies, including thyroid and parathyroid gland tests and serum levels of Ca, P were within normal limits. Tumor markers were within normal limits, including CA125, CEA, CA19.9, LDH, BhCG, Inhibin B and AFP. In the past medical history, the colonoscopy studies had shown no pathologic findings (performed due to 6-month history of constipation).

**Fig. 1:**
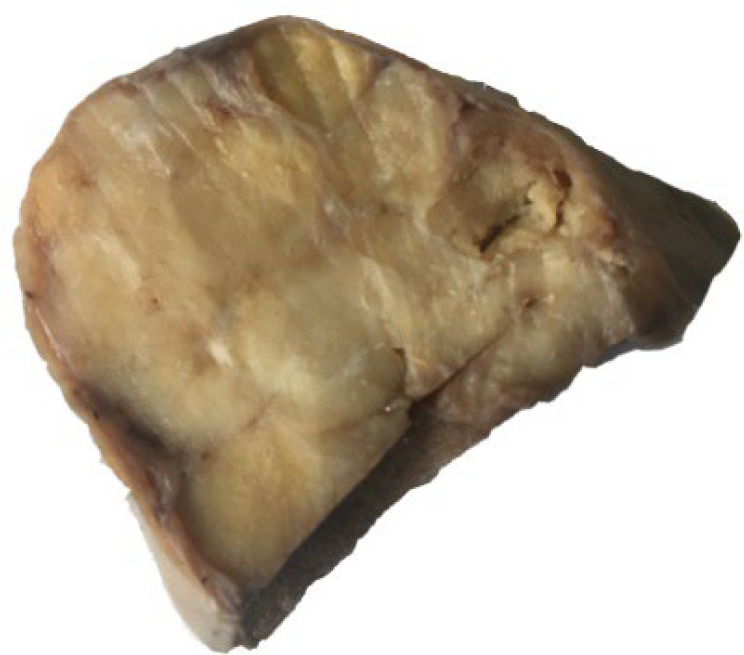
Postoperative view of the right adnexum revealed an encapsulated mass. Cut sections showed homogenous creamy - gray- yellow soft tissue with hemorrhage

**Fig. 2:**
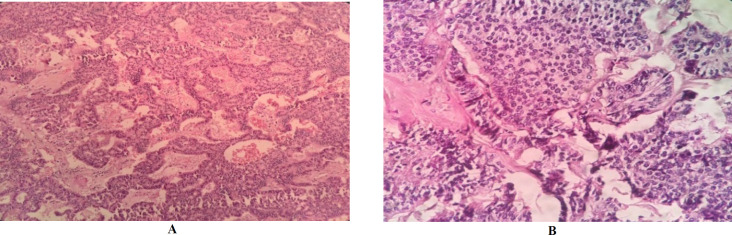
A: Histopathologic findings of ovarian neuroendocrine tumor in insular, trabecular, stromal and small acini growth pattern. B: Individual tumoral cells with neuroendocrine nuclear properties, eosinophilic cytoplasm and distinct cell borders. 100 (A), 400 (B), Hematoxylin & Eosin stain

During surgery, torsion of the right adnexal mass was observed, and the received specimen was an intact gray ovaloid mass in appearance, M: 6×5×5 cm (Figure 1). The cut sections showed homogenous, creamy, gray, and yellow soft tissue with cyst formation, hemorrhage, and a gritty sensation. Histopathology examination displayed ovarian encapsulated neoplasm in insular, trabecular, stromal, and small acini growth patterns. Individual monomorphic polygonal tumoral cells were seen, characterized by centrally located round to oval nuclei, salt and pepper chromatin, eosinophilic cytoplasm with distinct cell borders, with the mitotic activity of <2 mitoses/10 HPF. Microcalcifications were found (Figure 2 A, B), and no necrotic foci and capsular invasion were seen. On extensive sampling, the teratomatous elements are not found. The appendectomy specimen was unremarkable.

**Fig. 3:**
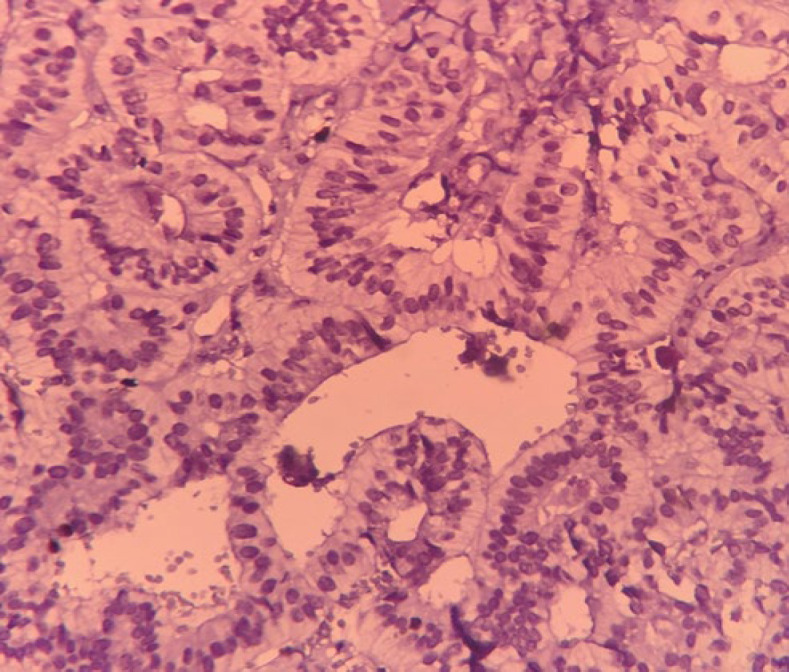
Nuclear immunoexpression of Ki67 in primary ovarian neuroendocrine tumor, < 3%. 400 , IHC stain

**Fig. 4.**
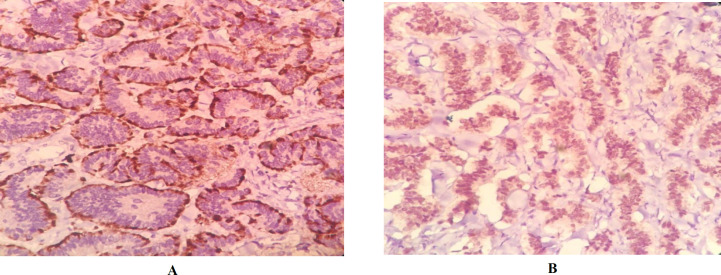
A: Granular cytoplasmic immunoexpression of chromogranin in primary ovarian neuroendocrine tumor. B: Nuclear expression of PAX8 in primary ovarian neuroendocrine tumor. 400 , IHC stain

The Ki67 index was less than 3%, and granular cytoplasmic immunoexpression of CgA and nuclear expression of PAX8 were detected (Figure 3 and 4 A, B). The surgical stage of the patient was determined as the FIGO 1, and histopathology and immunohistochemistry (IHC) result confirmed a mixed type of pure primary ovarian NET G1. Based on sonographic studies, there was no evidence of tumor recurrence during the 20-month follow-up.

## Discussion

We reported a new case of pure primary ovarian NET in a 26-year-old woman, a neoplasm emerging mostly in five or later decades of life and rarely in young adults (1, 2, 12). The main clinical symptom of the patient was an aggravating abdominal pain and also chronic progressive constipation. Less than 30% of patients with carcinoid tumors represent symptoms of carcinoid syndrome, making these tumors mostly an incidental finding (3, 7, 8). The most common causes of ovarian torsion include ovarian masses and cysts, specifically those of larger size (> 5 cm in diameter, especially larger than 10 cm) (13). In the present case, the possibility of torsion seemed low due to the low grade and relatively small tumor size. 

Constipation is a very uncommon symptom of carcinoid syndrome and is rarely seen in patients with ovarian NET (8, 14). Due to the presence of constipation as a nonspecific symptom, the patient's diagnosis was delayed until the abdominal pain became acute.

In imaging studies, ovarian NETs may appear in different forms, such as a pure form, a solid mass with small cysts, a nodule within a mature teratoma, a mucinous cystadenoma, or even within a Brenner tumor (9, 3). In the CT scan of our patient, a well-defined cystic-solid mass, measured 63 × 53 ×53 mm, and echogenic foci inside the solid part were seen. Despite the approximately small size of the tumor, our patient suffered from chronic progressive constipation, which seems to be caused by the hormonal effect of the YY peptide rather than the mechanical effect of the tumor. Similar to previous case reports of ovarian NETs (15), tumor markers were within normal limits in the present case.

The histomorphologic diagnosis of the mass was a mixed type of pure primary ovarian NET, known as a non-prevalent type (4). In this case, IHC studies suggested the ovarian origin of the tumor and the low grade of neuroendocrine tumors (5, 10, 11, 15). Based on the IHC results of the previous studies, CgA, ki67 index, synaptophysin, and CD56 are the most confirming markers to detect OCTs, and PAX8 can be used to distinguish them from a metastatic lesion (10, 11, 15). Due to the rare nature of ovarian NETs, serum levels of biologically active substances, including YY peptide and twenty-four-hour urine for 5-HIAA, were not initially evaluated. 

## Conclusion

Primary pure ovarian NET is a very rare type of ovarian neoplasms. The initial clinical presentations may be very diverse and unspecific, necessitating a complete assessment and follow-up of each patient, especially those with progressive symptoms, to make an early and reliable diagnosis. To exclude secondary ovary involvement, histopathologic diagnosis should be confirmed by the IHC studies. Today, it is challenging to establish diagnostic criteria for grading ovarian NETs. Data accumulation of more cases will indeed provide other experiences.

## Conflict of Interest

The authors declared no conflicts of interest.

## Sources of funding

 No source of funding.
